# Construction of an almond linkage map in an Australian population Nonpareil × Lauranne

**DOI:** 10.1186/1471-2164-11-551

**Published:** 2010-10-09

**Authors:** Iraj Tavassolian, Gholmereza Rabiei, Davina Gregory, Mourad Mnejja, Michelle G Wirthensohn, Peter W Hunt, John P Gibson, Christopher M Ford, Margaret Sedgley, Shu-Biao Wu

**Affiliations:** 1 School of Agriculture, Food and Wine, The University of Adelaide, Glen Osmond, SA 5005, Australia; 2 Centre for Genetic Analysis and Applications and School of Environmental and Rural Science, University of New England, Armidale, NSW 2351, Australia; 3 IRTA. Centre de Recerca en Agrigenòmica CSIC-IRTA-UAB. Carretera de Cabrils Km2. 08348 Cabrils (Barcelona), Spain; 4 CSIRO Livestock Industries, FD McMaster Laboratory, Chiswick, New England Highway, Armidale, NSW 2350, Australia; 5 Faculty of Arts and Sciences, University of New England, Armidale, NSW 2351, Australia; 6 Current Address: Faculty of Agriculture, University of Shahid Bahonar, Kerman, Iran; 7 Current Address: Agricultural Research & Development, Tasmanian Alkaloids, Westbury, TAS 7303, Australia

## Abstract

**Background:**

Despite a high genetic similarity to peach, almonds (*Prunus dulcis*) have a fleshless fruit and edible kernel, produced as a crop for human consumption. While the release of peach genome v1.0 provides an excellent opportunity for almond genetic and genomic studies, well-assessed segregating populations and the respective saturated genetic linkage maps lay the foundation for such studies to be completed in almond.

**Results:**

Using an almond intraspecific cross between 'Nonpareil' and 'Lauranne' (N × L), we constructed a moderately saturated map with SSRs, SNPs, ISSRs and RAPDs. The N × L map covered 591.4 cM of the genome with 157 loci. The average marker distance of the map was 4.0 cM. The map displayed high synteny and colinearity with the *Prunus *T × E reference map in all eight linkage groups (G1-G8). The positions of 14 mapped gene-anchored SNPs corresponded approximately with the positions of homologous sequences in the peach genome v1.0. Analysis of Mendelian segregation ratios showed that 17.9% of markers had significantly skewed genotype ratios at the level of P < 0.05. Due to the large number of skewed markers in the linkage group 7, the potential existence of deleterious gene(s) was assessed in the group. Integrated maps produced by two different mapping methods using JoinMap^® ^3 were compared, and their high degree of similarity was evident despite the positional inconsistency of a few markers.

**Conclusions:**

We presented a moderately saturated Australian almond map, which is highly syntenic and collinear with the *Prunus *reference map and peach genome V1.0. Therefore, the well-assessed almond population reported here can be used to investigate the traits of interest under Australian growing conditions, and provides more information on the almond genome for the international community.

## Background

Almond (*Prunus dulcis *(Mill) D. A. Webb) is an ancient plant species domesticated by humans initially in the Middle East. *P. fenzliana *or *P. communis *have been considered its most likely wild ancestors [1,2]. Valued for its health benefits and high nutritional value, the importance of the crop is increasing in the human diet, and consequently its production and commercial value are growing worldwide. Despite a high genetic similarity to peach, almonds have a fleshless fruit and edible kernel, rather than an edible fruit. Many agronomic traits important to almond such as shell hardness, kernel taste, kernel weight, resistance to biotic/abiotic stress, blooming time and self-incompatibility have been investigated, and efforts towards mapping and molecular characterisation of these genes have been made [3-13]. With the aid of the peach genome sequence released recently [14], characterisation of the almond genes responsible for agronomically important traits will become easier. However, well-assessed almond mapping populations and subsequent genetic maps are still fundamental for investigations of the genetic and molecular control of important traits.

A saturated linkage map can be a useful tool in the study of plant genetics and breeding. Close associations between important traits and molecular markers can assist fast selection of plants with desired features at early stages of growth. This is particularly valuable for breeding programs of woody plants because conventional, phenotype-based selection in these is delayed due to a long juvenile stage. Arùs *et al. *(1994) first reported a linkage map in a 'Ferragnes' × 'Tuono' (F × T) almond population with RFLP and isozyme markers, where the map distance was omitted [15]. Later the density and coverage of the map was improved by the addition of more markers [16,17]. Ballester (1998) constructed a molecular genetic map of a cross 'Felisia' × 'Bertina' (F × B) [18], and later a *Late bloom *gene was mapped in the population [13]. Sánchez-Pérez *et al. *(2007) mapped 11 traits (genes or QTL) in a cross of 'R1000' × 'Desmayo Largueta' (RxD) with 56 SSRs in the map [4]. However, more mapping populations and saturated maps are required to assist broader assessment of the almond traits and gene discovery especially under different environments and management systems. In Australia, a genetic linkage mapping program was initiated by Gregory [19] in a 'Nonpareil' × 'Lauranne' cross (N × L), but the integrated map with mainly RAPDs and ISSRs was sparse and further saturation was desirable [20]. Recently, Wu *et al. *(2009) reported that 12 SNP-anchored genes were mapped on six linkage groups in the same population with a higher map density [21]. Genetic maps have also been constructed in crosses between almond and peach including 'Texas'x'Earlygold' (T × E) [22-24], 'Garfi'x'Nemared' (GxN) [25], 'Padre' × '54P455' (Px5) [26,27] and a derivative population from GxN [28]. The most important map is the T × E map that has been generally accepted as a marker-saturated *Prunus *reference map. This map has been used to position genes corresponding to 1236 ESTs [29], locate 42 putative resistance regions [30], and align 613 rosaceaous unigenes that correspond to single copy *Arabidopsis *genes [31]: the Rosaceae Conserved Orthologous Set (RosCOS) map. A number of QTL have also been mapped using the T × E map [11].

In this study, we developed a moderately saturated linkage map by adding SSRs and SNPs to the N × L F1 map constructed by Gregory *et al. *(2005) [20]. The map was compared with the *Prunus *T × E reference map to demonstrate high synteny and colinearity between the N × L and the reference map. The sequences of the gene-anchored SNPs [21,32,33] were also compared to the peach genome v1.0 and the mapped positions generally agreed with the peach genome positions. The N × L genetic map reported here can be used to investigate traits of interest under the Southern Australian winter rainfall inland environment, and provides more almond genome information for the international community.

## Results

### Marker polymorphism

Altogether, 179 markers were polymorphic in the population under analysis. Of these, 92 (51.4%) were heterozygous in both parents, 34 with 4 alleles, 37 with 3 alleles, and 21 with 2 alleles; 40 (22.3%) were heterozygous only in Nonpareil; and 47 (26.3%) were heterozygous only in Lauranne. SSR markers BPPCT009, CPDCT020, CPSCT039 and UDAp-479 demonstrated multi-locus amplifications, each with two loci, across the population. The mapping results (see following description) indicated, however, that the loci of BPPCT009 were located closely in the same linkage group at a distance of 6.0 cM.

### Segregation of the markers

Of 179 markers analysed, 147 (82.1%) segregated in the expected Mendelian segregation ratios, and 32 (17.9%) showed skewed segregations (P < 0.05), with 19/113 (16.8%) SSR, 6/34 (17.6%) ISSR, 5/14 (35.7%) RAPD, and 2/14 (14.3%) SNP markers showing skewed ratios. Following grouping of the markers in the mapping process, 10/20 (50.0%) of the markers appearing in linkage group 7 (G7) had skewed segregation ratios, which was extremely high compared to the average across other groups 22/159 (13.8%). Interestingly, the only two skewed SNP markers were grouped in G7. To avoid using too few markers for a framework construction, all the skewed markers in G7 were included in the first step of mapping, and second step mapping for this group was omitted.

**Figure 1:**
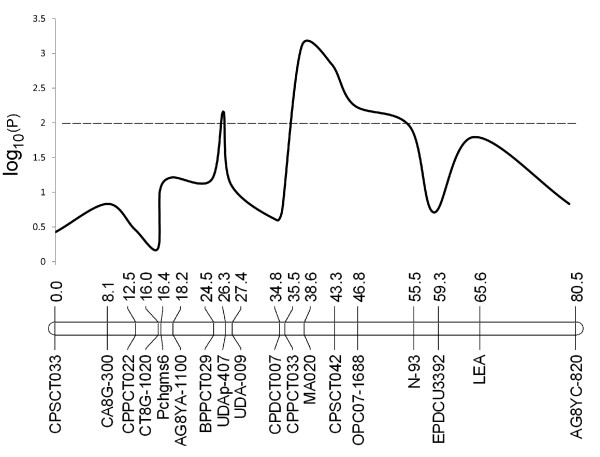
**A plot of negative log_10 _of *p*-values [-log_10_(*p*)] in χ^2 ^tests comparing frequencies of alleles of the loci in the G7 versus their map positions resulting from the One-step mapping method**. A main peak was shown in the area between markers CPPCT007 and N-93. A higher -log_10_(*p*) demonstrates the higher deviation of genotype segregation of a locus from the expected Mendelian ratio. Horizontal broken line shows threshold of statistical significance [-log10(*p*) = 2, corresponding to *p *= 0.01].

A plot of negative log_10 _of *p*-values [-log_10_(*p*)] in χ^2 ^tests comparing frequencies of alleles of the loci in the G7 versus their map positions is shown in Figure 1. A main peak was identified in the area between markers CPPCT007 and N-93. While a few markers with low -log_10_(P) values were present in the adjacent areas of the peak, a trend that the -log_10_(P) values declined gradually towards two ends of the linkage group was clearly illustrated.

### Linkage maps constructed using One-step and Two-step methods

A final linkage map of 591.4 cM containing 157 markers (93 SSRs, 35 ISSRs, 14 SNPs, 4 *S*-alleles, and 11 RAPDs) was constructed using the One-step method of JoinMap^® ^(Table 1). The average marker distance of the map was 4.0 cM, and 27 skewed markers were mapped in the genome of the population. Individually, G1 was the longest group covering 108.2 cM with 22 markers. G5 was the shortest group covering 54.8 cM with 17 markers. The average marker distance varied from 2.4 (G6) to 5.7 (G8) cM. As indicated previously, G7 mapped the highest number of segregation ratio skewed markers (10), whereas G5 had no skewed markers in the group.

**Table 1:** Statistics of the maps constructed using One-step and Two-step methods.

One-step					Two-step			
	
Group	Number of loci	Size (cM)	Average marker distance (cM)	Number of skewed markers	Number of loci	Size (cM)	Average marker distance (cM)	Number of skewed markers
G1	22	108.2	5.2	3	24	113.0	4.9	3
G2	16	56.0	3.7	1	16	48.4	3.2	1
G3	21	69.4	3.5	5	21	72.3	3.6	6
G4	21	83.6	4.2	3	21	89.4	4.5	3
G5	17	54.8	3.4	0	17	54.4	3.4	0
G6	28	65.0	2.4	2	28	72.8	2.7	2
G7	18	80.5	4.7	10	18	81.6	4.8	10
G8	14	73.9	5.7	3	15	76.5	5.5	3
**Total/average**	**157**	**591.4**	**4.0**	**27**	**160**	**603.9**	**4.0**	**28**

Using the Two-step method, the final map was 603.9 cM contained 160 markers (95 SSRs, 35 ISSRs, 18 SNPs/INDELS, and 12 RAPDs) (Table 1). The average marker distance of this map was 4.0 cM and 28 skewed markers were included in the map. Similar to the One-step map, G1 was the longest group at 113 cM containing 24 markers. G5 was the shortest group at 54.4 cM containing 17 markers. The average marker distance varied from 2.7 (G6) to 5.5 (G8). As with the One-step method, G7 had the highest number of skewed markers (10) whereas G5 had no skewed markers mapped.

**Figure 2:**
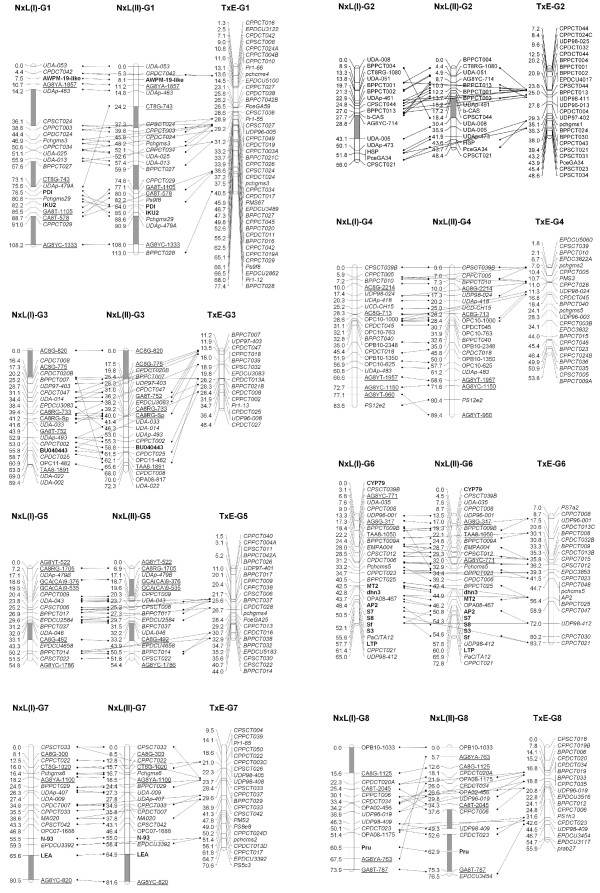
**The alignment of the linkage groups between maps of the cross 'Nonpareil' × 'Lauranne' (N × L) F1 population constructed using One-step and Two-step methods, and with the *Prunus *T × E reference map that include only SSR markers**. The maps of N × L population were constructed using the JoinMap^® ^3, and the maps were viewed and aligned by the MapChart 2.2. The SSR markers are shown in *italics*, the SNP-anchored genes and S-loci are in **bold**, ISSR markers are underlined, and the RAPD markers are in plain font. The genetic distance of the loci are shown in centimorgans (cM) and the gaps between two adjacent markers > 10 cM are highlighted in grey segments on the linkage group bars.

For most of the markers, the two methods produced consistent mapping results as shown in Figure 2. The linkage groups G5 and G7 produced by the One-step and Two-step methods were completely collinear with no rearranged linkage order. Other linkage groups had one or more markers in different map order in the two maps. Although the divergence in positions were not substantial for most markers, seven markers showed shifts larger than 20 cM, i.e., CT8G-743 in G1, UDA-008 and AG8YC-714 in G2, CPDCT008 in G3, AG8YC-771 in G6 and AG8YA-763 and OPA08-1175 in G8 with shifts of: 48.9 cM, 30.4 cM, 20.1 cM, 41.6 cM, 25.2 cM, 61.8 cM, and 29.5 cM respectively. Among these, CT8G-743, CPDCT008, AG8YC-771 and AG8YA-763 showed significantly skewed segregation ratios, and most (5/7) were dominant markers (ISSR and RAPD). The segment from marker UDAp-479A to marker CPPCT029 of G1 (in map I) and from marker CA8T-2045 to marker UDP96-019 of G8 (in map I) was inverted between the two maps.

Among the markers mapped in the N × L population, the ISSRs were developed in our initial analysis, and therefore were newly mapped markers in the *P. dulcis *map. The SSRs were identified from published studies, and the majority of the markers mapped in the same linkage groups as previously reported. However, a few SSR markers were mapped for the first time: CPDCT018 on G4, CPDCT006 on G6, and CPDCT007 on G7. Moreover, some markers amplified more loci in the N × L population than in the previous report or mapped to different linkage groups. CPDCT020 mapped to G8 in the T × E reference map [23] and the "Contender" × "Fla.92-2C" peach map [34] as a single locus marker. The primers for this marker amplified two loci in the N × L population, which mapped to G3 and G8. Primers for UDAp-479 also amplified two loci, which mapped to G1 and G5 in our study. A recent report identified four loci for UDA-479 in the apricot population "Z506-07" (Z) × "Currot" (C) and all four loci were assigned to G8 [35]. Two loci of BPPCT009 were mapped to G4 of the peach "Ferjalou Jalousia" × "Fantasia" [36] and T × E reference maps [23] for locus A, and G7 of the "Ferjalou Jalousia" × "Fantasia" map [36] and G6 of the T × E reference map [23] for locus B. In contrast, two loci for BPPCT009 were mapped to G6 of our mapping population in an interval of 6.0 cM.

In the maps constructed by both methods, gaps bigger than 10 cM were observed (segments in grey shown in Figure 2). These included one gap on G2, G3, G5, G7 and G8, and three gaps on G1 in the One-step map, and one gap on G2, G3, and G7, two gaps on G5, and three gaps on G1 and G8 in the Two-step map. The Two-step map had more gaps (11) of > 10 cM than the One-step map (8). The biggest gap (21.9 cM) was between the markers UDAp-463 and CPSCT024 on G1 of the One-step map.

### Synteny of the N × L and T × E maps and between the almond and peach genomes

The almond N × L and *Prunus *T × E reference genetic maps were compared using common SSR markers to visualise the syntenic regions. As shown in Figure 2, a high degree of macro-synteny between N × L and T × E was evident across the whole genome with 59 common SSR markers. For example, the linkage groups G1, G4 and G6 did not show any order conflict between the N × L and T × E maps. Despite the high degree of macro-synteny, rearrangements of markers in small sections occurred in the other linkage groups. Furthermore, a few markers showed inconsistency of position over larger distances between the N × L and T × E maps. For instance, the marker CPSCT033 mapped to the top of G5 in the N × L maps (I and II), while it was located in the middle segment of T × E 28.4 cM from the top. The marker CPDCT008 was mapped to the upper part (N × L map I) or the lower part (N × L map II) of G3 but to the lower middle part in T × E. With reference to the T × E map, the N × L map coverage of the genome varied with linkage groups. G1 and G6 covered the whole length of the corresponding groups of T × E; G2, G3, and G7 covered most of their corresponding groups with one end or both ends having no common markers with T × E but covering equivalent lengths; G4 and G5 had fewer markers in common with the T × E map but comparison with maps in the GDR database http://www.rosaceae.org[37] indicated full coverage of G4; and G8 alignment indicated that at least the bottom part of approximately 10 cM was not mapped in N × L. Hence, this N × L map can be regarded as moderately saturated.

**Figure 3:**
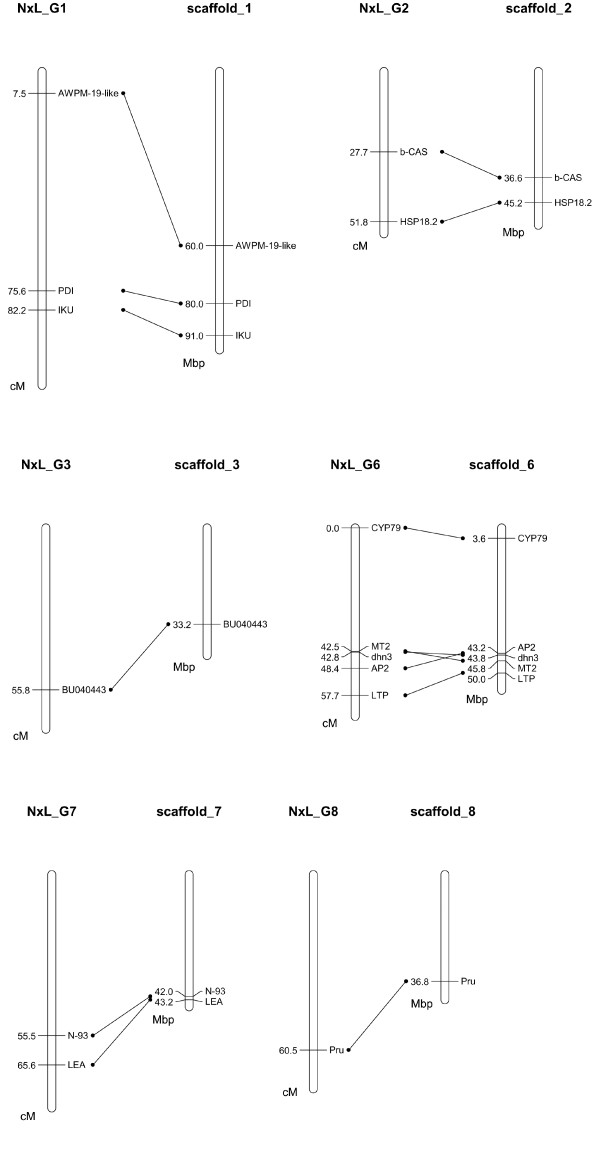
**Comparison of the positions of SNP-anchored genes mapped in the N × L population (map I) with the positions of their homologous sequences in the scaffolds of peach genome v1.0**. The genetic distance in the N × L map is shown in centimorgans (cM), and the positions of the homologous sequences in the scaffolds of peach genome v1.0 are shown in mega basepairs (M). The scaffold bars represent the whole length of the corresponding chromosomal DNA. The locations of all the genes except AWPM-19-like mapped in the N × L population agreed with the positions of their homologous sequences in corresponding peach genome scaffolds. AWPM-19-like gene positioned near the top of N × L G1, but in the lower part of the peach genome scaffold_1. An inversion is present in the segment including genes MT2, dhn3, and AP2.

The sequences of fourteen SNP-anchored genes were compared using Blastn with the peach genome v1.0 database, and homologous sequences were located in the scaffolds of the peach genome. As the scaffolds correspond to each of the linkage groups of *Prunus *maps, the relative positions of the genes can be identified in the genome. The results showed that the locations of the majority of the genes mapped in the N × L population agreed with the positions of their homologous sequences in the corresponding peach genome scaffolds (Figure 3). AWPM-19-like, however, was located near the top of G1 rather than in the lower middle part of the group, where the homologous sequence was identified in the peach genome scaffold_1. In G6, the segment involving MT2, dhn3 and AP2 showed inversion compared to the peach genome despite spanning only a small fragment with genetic distance of 5.9 cM or DNA length of 2.6 Mbp.

## Discussion

In this study, we constructed almond linkage maps of an Australia population derived from the cross between the American cultivar 'Nonpareil' as maternal parent and the French cultivar 'Lauranne' as pollen donor (N × L). Two maps were constructed using One-step and Two-step methods, with total lengths of 591.4 cM and 603.9 cM respectively. 157 molecular markers were positioned on the One-step and 160 markers were positioned on the Two-step map. The resulting maps showed high colinearity with the *Prunus *T × E reference map [24,36].

To obtain an integrated map of a cross pollinated population, individual parental maps were generally constructed and then integrated to produce a consensus map of the population by estimation of the average recombination frequency of the loci in the two parents, as has been used for other pseudo-test cross mapping populations in many tree species [38-40]. In this study, we denoted this as the Two-step method. Since the release of JoinMap^® ^version 3 [41], the construction of an integrated map of the population can be undertaken by loading all the genotyping data of the population, bypassing individual parental map construction in a One-step strategy. Genetic maps constructed in this way have been published in recent years [42-44]. During initial mapping analysis, we tried both the One-step and Two-step methods, and variations of marker distances and positions were identified. As the comparison studies had not been reported, we applied both methods in our N × L almond population to investigate whether different methods yield significantly different maps. Based on our study, these two integration methods did not result in substantial differences for all the linkage groups, and only a small proportion of markers showed positional instability between the two maps. The majority of the markers (6/7) that had a position shift of greater than 20 cM between two maps showed skewed segregation ratios or were adjacent to markers with skewed segregation. The fewer large gaps generated in the One-step map suggest that the One-step mapping is an appropriate method to construct an integrated map of a pseudo-test cross population such as in the almond and other tree plants. Therefore, in this study, the One-step map was used to represent the N × L genome for other analyses (Figures 1 and 3).

Linkage maps based on intraspecific crosses of almond have been reported for the crosses of 'Ferragnes' × 'Tuono' (F × T) [15-17], 'Felisia' × 'Bertina' (F × B) [13,18], 'R1000' × 'Desmayo Largueta' (RxD) [4], and 'Nonpareil × Lauranne' (N × L) [20,21]. While F × T and F × B maps consisted mostly of RFLP and RAPD markers, the RxD map contains 56 SSR markers with less density across the genome. SSRs are the favoured marker type used for many applications in plant genetics including genetic mapping because of easy transferability between intraspecific populations and across closely related species, and a high number of alleles per locus that provides greater information content [45-48]. Therefore, a saturated map containing additional SSR markers is warranted in almond intraspecific crosses. The N × L map was initiated with RAPDs, ISSRs and the small numbers of SSR, and a sparse integrated map was subsequently produced [20]. Using high resolution melting curve analysis [32], Wu *et al. *mapped 12 gene-anchored SNPs on six linkage groups plus the addition of more SSR to the map [21]. In the present study, we have constructed a combined molecular linkage map including SSR, SNP, RAPD, and ISSR markers. In comparison with the *Prunus *T × E reference map and other maps reported in *Prunus *(data not shown), the linkage groups of the N × L map covered close to the whole length of the almond linkage groups with the exception of G8 which requires further extension beyond EPDCU3454. With reasonable dense coverage of the genome by SSRs and SNPs, the map can readily be used in the Australian almond breeding program [49] and contribute to international almond genome research.

The clustering of loci with skewed segregation ratios on G7 suggests a possible association of deleterious genes with this section of the linkage group and a more in depth study to investigate this possibility is warranted. In a peach F2 mapping population, no linkage of markers could be established for G7 [50] possibly due to the complexity of marker segregation. In our study, more than half (10/18) of the mapped markers had significantly skewed segregation ratios, and most of the skewed markers clustered on the central section of the linkage group with a peak of segregation distortion around marker MA020. This finding indicates that the area may harbour one or more deleterious genes. Although some genes or traits related to biotic or abiotic stress have been mapped to this group such as the nematode resistance trait MA [30] and the DHN gene involved in freezing and drought tolerance [27], those genes were probably not the cause of the distorted segregations as these occur in a different region of G7. It would be interesting to search for deleterious gene alleles in the region around MA020. The recent release of peach genome v1.0 provides a good opportunity for conducting such investigations.

With the release of a >7 fold coverage peach genome in April 2010 (v1.0), with 27,852 genes predicted [14], genomic exploration in *Prunus *and more widely in the family Rosaceae and perhaps other tree plants will accelerate. In the present study, we compared 14 SNP-anchored genes mapped in the N × L population with the peach genome v1.0. A high synteny between our map and the peach genome was observed as expected. However, an inversion was noted in a G6 segment including genes MT2, dhn3, and AP2. The evidence for the inversion will become clearer when the almond genome is sequenced and a final sequence build is achieved in the future. Nevertheless, as the closest relative of peach, genetic and genomic studies in almond will benefit significantly from the publication of the peach genome sequences prior to the complete sequencing of the almond genome. For almond researchers and breeders to fully utilise the sequence information becoming available for peach, well-assessed almond populations and genetic maps are required to associate important agronomic traits of the species with predicted genes in peach. Development of saturated genetic marker maps such as that presented in this paper will be valuable for almond genetic research and breeding programs.

## Conclusions

Here, we presented a moderately saturated Australian almond map, which is highly syntenic and collinear with the *Prunus *T × E reference map and peach genome V1.0. It was identified that a section of G7 with skewed markers may harbour one or more deleterious gene(s), and further investigation to search for such a gene is suggested. The comparison of One-step and Two-step methods indicated that these two methods produced highly consistent maps, but One-step method was a preferred mapping approach. The well-assessed almond population reported here can be used to investigate the traits of interest under Australian growing conditions, and provides more information on the almond genome for the international community.

## Methods

### Mapping population and DNA extraction

An almond pseudo-testcross population with 93 progeny, derived from the cross between the American cultivar 'Nonpareil' as maternal parent and the French cultivar 'Lauranne' as pollen donor, was used as the mapping population (N × L) [21]. The population was planted in a commercial orchard at Lindsay Point - Victoria, Australia (34°15'27"S - 141°00'00"E) with a fertile and well drained soil and an average of 223 mm annual rainfall. Standard orchard management including fertilisation, irrigation and pruning were applied. Total genomic DNA was extracted from fresh young leaves using the protocol of Lamboy and Alpha, (1998). DNA quantity and quality was measured spectrophotometrically by Nanodrop ND-1000^® ^(Thermo Scientific, USA).

### Molecular markers

A total of 241 SSR initially reported in different *Prunus *species were screened for polymorphisms in the parents and selected progeny (Table 2). The designation of the markers, the original species from which the markers were developed, and the reference information are listed in Table 2. The PCR was performed in a total volume of 20 μl containing 1 × PCR reaction buffer (Bioline, Sydney, Australia), 2.5 mM MgCl_2_, 0.2 mM dNTPs, 0.2 μM of each primer, 40 ng of template DNA and 1 unit of *Taq *polymerase (Bioline, Sydney, Australia). Amplification involved first denaturation at 95°C for 5 min, 34 cycles of denaturation at 95°C for 30 seconds, annealing at appropriate temperatures (mostly based on the information provided in the cited literature, and available on request from authors) for 30 seconds, and extension at 72°C for 30 seconds, and a final extension at 72°C for 7 min. Electrophoresis was performed on 8% (w/v) polyacrylamide gel, or automated capillary gel on the ABI PRISM 3730 DNA Analyzer (Applied Biosystems) to visualise PCR products. Markers with good reproducibility and clearly decipherable loci were chosen for construction of linkage maps.

**Table 2:** List of identifiers and numbers of the SSR markers tested, segregated and mapped.

Identifier	Number of markers tested	Number of markers segregated	Number of markers mapped	Species of origin	References
AMPA	1	0	0	Apricot	[52]
BPPCT	29	17	16	Peach	[53]
CPDCT	25	15	13	Almond	[47]
CPPCT	25	13	12	Peach	[54]
CPSCT	23	13	10	Japanese plum	[55]
EMPA	3	1	1	Sweet cherry	[56]
EMPaS	4	0	0	Sweet cherry	[57]
EPDCU	14	6	5	Almond	[48]
MA	1	1	1	Peach	[58]
Pac	2	0	0	Apricot	[52]
PaCITA	1	1	1	Apricot	[59]
PceGA	3	1	1	Sour cherry	[60]
Pchcms	3	1	1	Peach	[61]
Pchgms	8	3	3	Peach	[61,62]
PMS	3	0	0	Peach	[63]
PS	4	2	2	Sour Cherry	[16]
UCD-CH	8	2	1	Sweet cherry	[64]
UDA	41	15	14	Almond	[65]
UDAp	28	9	9	Apricot	[66]
UDP	15	8	6	Peach	[67]
**Total**	**241**	**108**	**96**		

The design and assay techniques for the SNPs, ISSRs and RAPDs used in the present study have been described previously [19-21,33]. The assay of self-incompatible genes *S3*, *S7*, and *S8*, and self-fertile gene *Sf *was conducted as described by Channuntapipat [6].

### Map construction

Linkage maps were constructed using JoinMap^® ^3 software [41]. Two different mapping methods were applied and the resulting maps were compared and analysed to assess their synteny with the *Prunus *T × E reference map. The first method constructed two parental maps separately, which was followed by the production of an integrated map. As this approach involved two map construction steps [38-40], we denoted it as 'Two-step method' and the map or linkage groups labelled as "II". This included preparing two separate parental data sets as described elsewhere for a pseudo-testcross population [38]. Both sets of genotyping data were loaded into JoinMap^® ^3 and the two parental maps were constructed separately. Chi-square analysis was performed for goodness of fit to the expected Mendelian segregation ratio for each marker and skewed markers were identified using a threshold of P < 0.05. Framework linkage groups were created by omitting the skewed markers from the data for all the linkage groups except G7, due to the high degree of skewed markers in this group (see Results section). These framework groups were used as fixed orders for the individual final map construction that included all markers. Linkage groups were established at a LOD score > 5 and recombination fraction < 0.40. The Kosambi mapping function was used for the calculation of map distances. Two parental maps (as frameworks or final maps) were integrated using the "Combine Groups for Map Integration" function of Joinmap^® ^3 to produce the combined maps (framework or final maps). This method uses mean recombination frequencies and combined LOD scores for mapping calculations. The second method constructed a map by using all the markers heterozygous in both or either of the parental trees as one set of data [42-44]. As this method involved using all markers in a single map construction, it was denoted as the 'One-step method' and the map or linkage groups labelled as "I". The process of testing segregation ratios and inclusion of markers in maps was identical to that used in the 'Two-step method'. Markers in common between our maps and the *Prunus *T × E reference map were used to identify corresponding linkage groups. The resulting maps were graphically presented and their alignment was performed using Mapchart 2.2 [51].

### Sequence blast and localisation in *Prunus *genome

Sequences of the SNP-anchored genes were blasted against peach genome v1.0 scaffolds [14], and the resulting homolog sequences were located in the scaffolds (corresponding to the linkage groups of *Prunus *genetic maps) using the GBrowse function http://www.rosaceae.org/gb/gbrowse/prunus_persica/.

## Authors' contributions

IT and GR performed SSR genotyping, mapping analysis, and interpretation of the data. DG conducted RAPD, ISSR and SSR genotyping and initial mapping analysis. MM participated in the SSR genotyping and mapping analysis. MGW participated in the design of the study, collected samples and managed the mapping population. PWH and CMF participated in the design of the study and revised the manuscript critically. JPG participated in the design of the study and mapping analysis, and revised the manuscript critically. MS conceived of the study, participated in its design and coordination and revised the manuscript critically. SBW participated in the design of the study, performed SNP genotyping, analysed SSR genotyping data, completed final mapping analysis, conducted sequence and map alignments and drafted the manuscript. All authors have read and approved the final manuscript.
